# Machine Learning Strategies When Transitioning between Biological Assays

**DOI:** 10.1021/acs.jcim.1c00293

**Published:** 2021-06-21

**Authors:** Staffan Arvidsson McShane, Ernst Ahlberg, Tobias Noeske, Ola Spjuth

**Affiliations:** † Department of Pharmaceutical Biosciences and Science for Life Laboratory, Uppsala University, 751 24 Uppsala, Sweden; ‡ Stena Line Scandinavia AB, AI & Data, 405 19 Gothenburg, Sweden; ¶ Imaging and Data Analytics, Clinical Pharmacology & Safety Sciences, R&D, AstraZeneca, 431 50 Gothenburg, Sweden; § Predictive Compound ADME & Safety, Drug Safety & Metabolism, AstraZeneca IMED Biotech Unit, 431 50 Gothenburg, Sweden

## Abstract

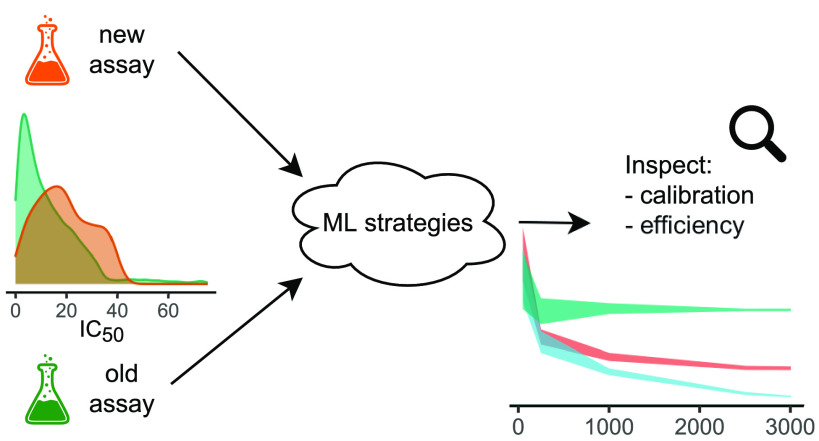

Machine learning is widely used in drug development to predict activity in biological assays based on chemical structure. However, the process of transitioning from one experimental setup to another for the same biological endpoint has not been extensively studied. In a retrospective study, we here explore different modeling strategies of how to combine data from the old and new assays when training conformal prediction models using data from hERG and Na_V_ assays. We suggest to continuously monitor the validity and efficiency of models as more data is accumulated from the new assay and select a modeling strategy based on these metrics. In order to maximize the utility of data from the old assay, we propose a strategy that augments the proper training set of an inductive conformal predictor by adding data from the old assay but only having data from the new assay in the calibration set, which results in valid (well-calibrated) models with improved efficiency compared to other strategies. We study the results for varying sizes of new and old assays, allowing for discussion of different practical scenarios. We also conclude that our proposed assay transition strategy is more beneficial, and the value of data from the new assay is higher, for the harder case of regression compared to classification problems.

## Introduction

Assessing properties of novel compounds using one or several biological and biochemical assays is a common methodology in preclinical drug discovery.^1^ Important properties include on- and off-target effects, ADME (Absorption, Distribution, Metabolism, Excretion) and Toxicity, and there exist a large number of assays developed for these and other endpoints.^2^ Predicting the result of an assay using in silico methods such as Machine Learning (ML), prior to performing the assay or even before synthesizing the compound,^3−5^ has increased in popularity over the years. When the chemical structure is used to represent the compound in such ML modeling, the method is referred to as QSAR or SAR (Quantitative Structure–Activity Relationships)^6^ and falls under what is called ligand-based methods. QSAR has been used to model a wide range of endpoints, such as interaction with various targets.^7,8^ The database ChEMBL^9^ collects a great deal of curated data from different compounds and assays, and it is a common approach to merge data for the same target from different assays into a single data set that is subjected to ML modeling.^10,11^

When evaluating the accuracy of a model, the standard protocol is to split the data set in a *training set* for training the model and a *test set* to evaluate its accuracy. There are also methods such as cross-validation that can be used to produce a balanced accuracy measure. The accuracy of QSAR models typically depends on the number of compounds/experiments in the training set. In pharmaceutical companies, the data generating process leads to a continuous expansion of assay data and hence over time increases the accuracy of their models. However, sometimes the organization might want to switch to another experimental setup to report on the same endpoint; this might be due to better capture the underlying phenomenon, decrease variance, or to reduce time and cost. Before transitioning to a new assay, it is common to run a set of compounds with both the old and the new assay in order to study the agreement between the measurements, referred to as *assay concordance*.^12,13^ These sets typically contain well characterized tool compounds covering the biological phenomena of interest (e.g., mode of actions) and compounds that are chemically diverse and span a wide potency range. Assay concordance may be determined by a statistical approach that analyzes the mean difference of both assays and produces an agreement interval accounting for 95% of the differences between both assays.^14^ However, how to use data from the old assay as efficiently as possible in downstream ML applications leads to several questions: (1) When the organization starts to generate data from the new assay, how should the data from the old assay be used; (2) Should models be trained exclusively on the new assay data, potentially resulting in low accuracy until a sufficient number of experiments have been run; (3) Should data from the old assay be pooled with the new assay, even though there is a known difference between them.

A core assumption of all ML methods is that the data used for training the model is *i.i.d.*, i.e., independent and identically distributed. If data from, e.g., an old assay and a new assay stem from different distributions, then a model trained on pooled data from both assays might not be valid and predictions cannot necessarily be trusted. There are methods devised to detect violations against i.i.d., commonly called data set shifts,^15,16^ but these are restricted to specific versions of shifts (e.g., covariate shift or concept shift). Several methods have also been proposed to increase the accuracy of the trained model when knowing that a data set shift is present.^17−20^

Conformal prediction (CP) is a mathematical framework developed for ML with the objective to produce well-calibrated predictions where the predictions adhere to a user-defined *confidence* (e.g., requiring 80% confidence results in at least 80% accurate predictions).^21,22^ CP assumes *exchangeability* between all data, which is a similar but a slightly weaker assumption than i.i.d. A benefit of using CP is that the calibration of test data can be inspected, and poor calibration can intrinsically reveal data shifts and improper handling of data. In recent work, we assessed CP for improving the calibration when a data drift has occurred and concluded that updating the calibration set improved calibration across all evaluated data sets.^23^ CP has been used extensively in various drug discovery applications.^24−29^

In this manuscript, we perform a retrospective analysis of how old data can be used most efficiently when a decision has been made to switch to a new assay system, using data from the hERG and Na_V_ endpoints at AstraZeneca. We refer to this specific problem as Assay Transition, and a distinguishing property of the problem is that it includes a continuous decision making process during the transition from one specific assay to another. We apply conformal prediction which enables us to evaluate the level of calibration and efficiency for different modeling strategies and discuss their implications.

## Materials and Methods

### Data

**Figure 1:**
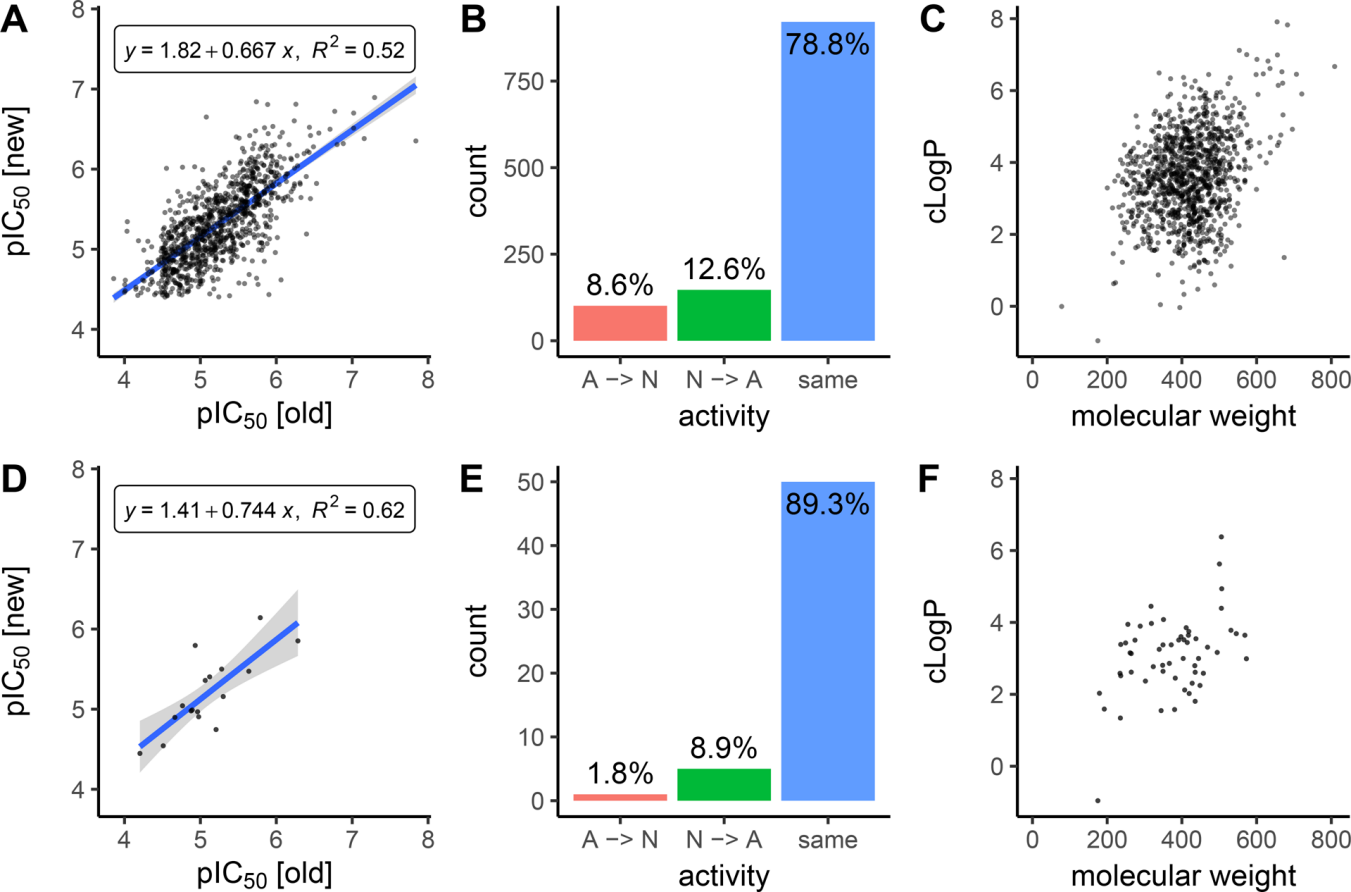
Analyzing compounds measured in both the old and new assays. The top panels show the overlap in hERG (1,169 compounds), and the bottom panels show Na_V_ (56 compounds). Panels A and D plot new measurements vs old measurements in pIC_50_, requiring exact assay values without qualifier, cutting down the overlap to 972 and 17 compounds, respectively. Fitting a linear model between old and new endpoint values (equations shown in the panels) indicate a positive correlation between new and old assays but with a slope far from the ideal (slope of 1). For hERG, the measurements were statistically different using a paired *t*-test (*p* = 1.95 × 10^–9^). The Na_V_ overlap, only containing 17 compounds, was too small to prove statistically different. Panels B and E display the change in categorical activity, when applying a 10 μM threshold. Both data sets retain the same activity-class for the majority of the compounds. Finally, panels C and F plot the computed logP (cLogP) versus molecular weight, with no apparent clusters in this “chemical space” and indicate that all compounds originate from druggable chemical space.^34^

In-house bioassay data sets from AstraZeneca were used, containing dose–response data for the two ion channels hERG and Na_V_.^30,31^ These are routinely screened in the early phases of drug discovery as they are tightly linked to cardiovascular risks^32^ and are thus among the largest data sets generated from single assays. The raw, unprocessed data sets contained in excess of 152,000 and 16,000 records for hERG and Na_V_, respectively. Some records included a qualifier (“>” or “<”), indicating that the endpoint value was not determined exactly but that the IC_50_ value was either larger or smaller than the tested concentrations. From late 2016 until early 2017, the in-house routine voltage-gated ion-channel assays were moved from the existing medium-throughput electrophysiology IonWorks^33^ device to the high-throughput SyncroPatch 384 PE platform. This switch facilitated technical improvements (reduced screening turnaround time, increased capacity, reduction of consumables spent) as well as the ability to detect slow onset ion-channel blockers. To assess how well the assays are agreeing, compounds tested in both assays were studied (Figure 1). Overall, 78.2% of hERG and 89.3% of Na_V_ compounds retained their categorical label after the transition (assuming a 10 μM threshold, see Data Preparation), see Figure 1B,E. Considering the measured endpoint values, the measurements for hERG were statistically different (*p* = 3.06 × 10^–12^ for IC_50_ and *p* = 1.95 × 10^–9^ for pIC_50_, using paired *t*-tests). Na_V_ only contained 17 compounds with exact assay values, too few to give a statistical difference, albeit visually there is a large variance in assay measurements in Figure 1D.

### Data Preparation

Data was acquired in CSV format including compound ID, signature feature counts^35^ using heights 1–3, test date, measured endpoint value, an optional qualifier (“>” or “<”), and some additional descriptors such as molecular weight and computed LogP. The qualifier indicates whether the endpoint value was determined to be that exact value, or if the IC_50_ value was either larger or smaller than the tested concentrations. A 10 μM threshold was used for categorizing compounds as active (A) or nonactive (N), according to ref (36). We define *A*_new_ to represent a data set containing observations from the new assay and *A*_old_ to represent the equivalent for observations from the old assay; the actual number of observations depends on the context. The data preparation steps are outlined in Box 1.
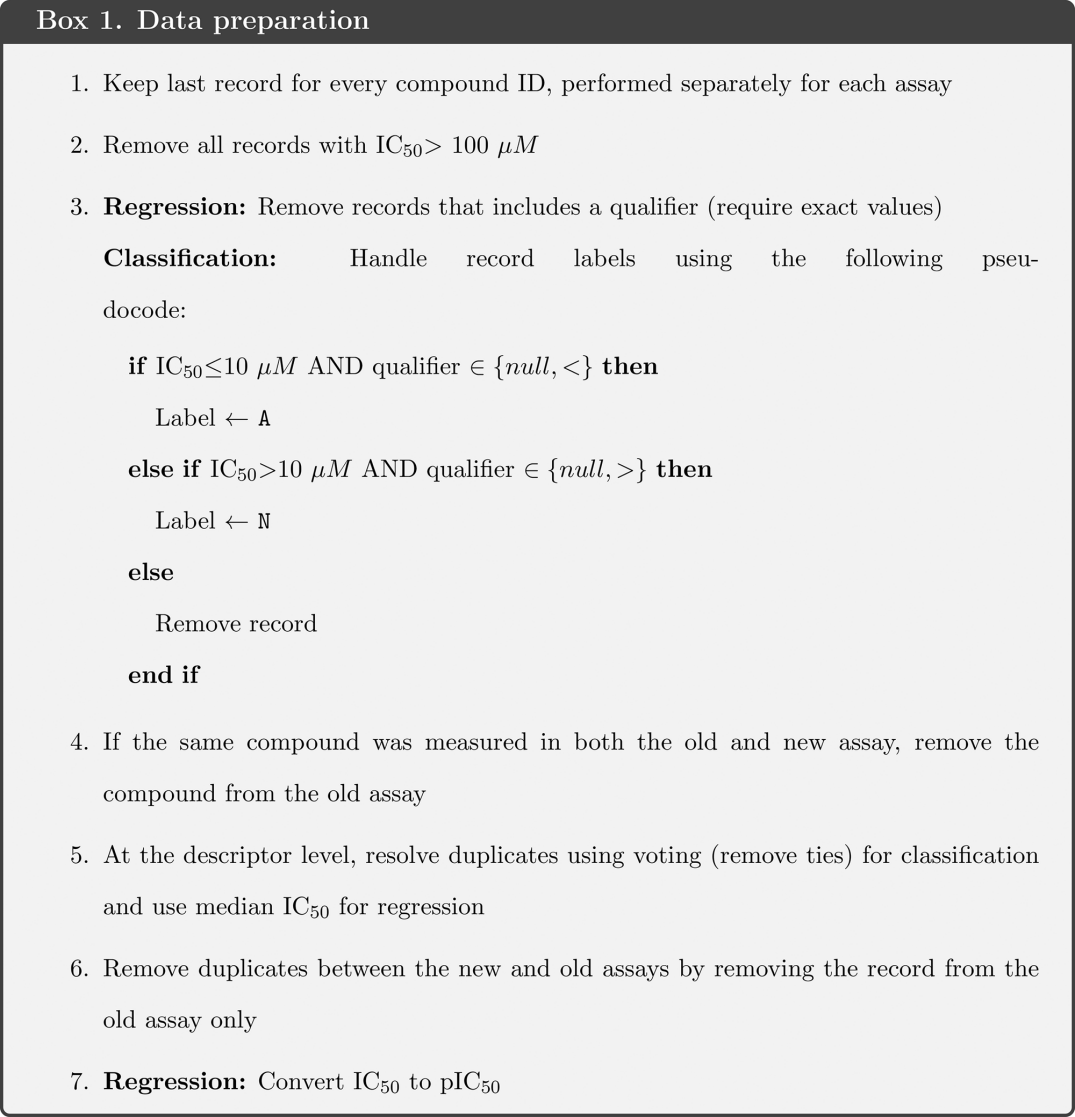


For the Na_V_ data set, step 2 also included removing all records with an IC_50_ of 33.3 μM, as it was found to be over-represented in the data set and likely an artifact. The exclusion of compounds recorded at over 100 μM was due to the typical experimental range was only up to 100 μM. Too few active compounds, i.e., with IC_50_ ≤ 10, were measured in the new Na_V_ assay, so the classification data set was excluded for further analysis. The size of the final data sets used in the analysis is found in Table 1, and the total number of signature descriptors was in excess of 27,000 (Na_V_), 80,000 (hERG regression), and 120,000 (hERG classification).

**Table 1:** Final Data Sets Used in the Experiments, after Filtration Steps and Applying the 10 μM Threshold for Generating Categorical Labels^a^

data set	*A*_new_	*A*_old_	% active [new]	% active [old]
hERG classification	4,800	64,000	45%	30%
hERG regression	3,300	36,400		
Na_V_ regression	190	4,900		

a Note that the final sizes are slightly rounded off for the confidentiality of AstraZeneca.

**Figure 2:**
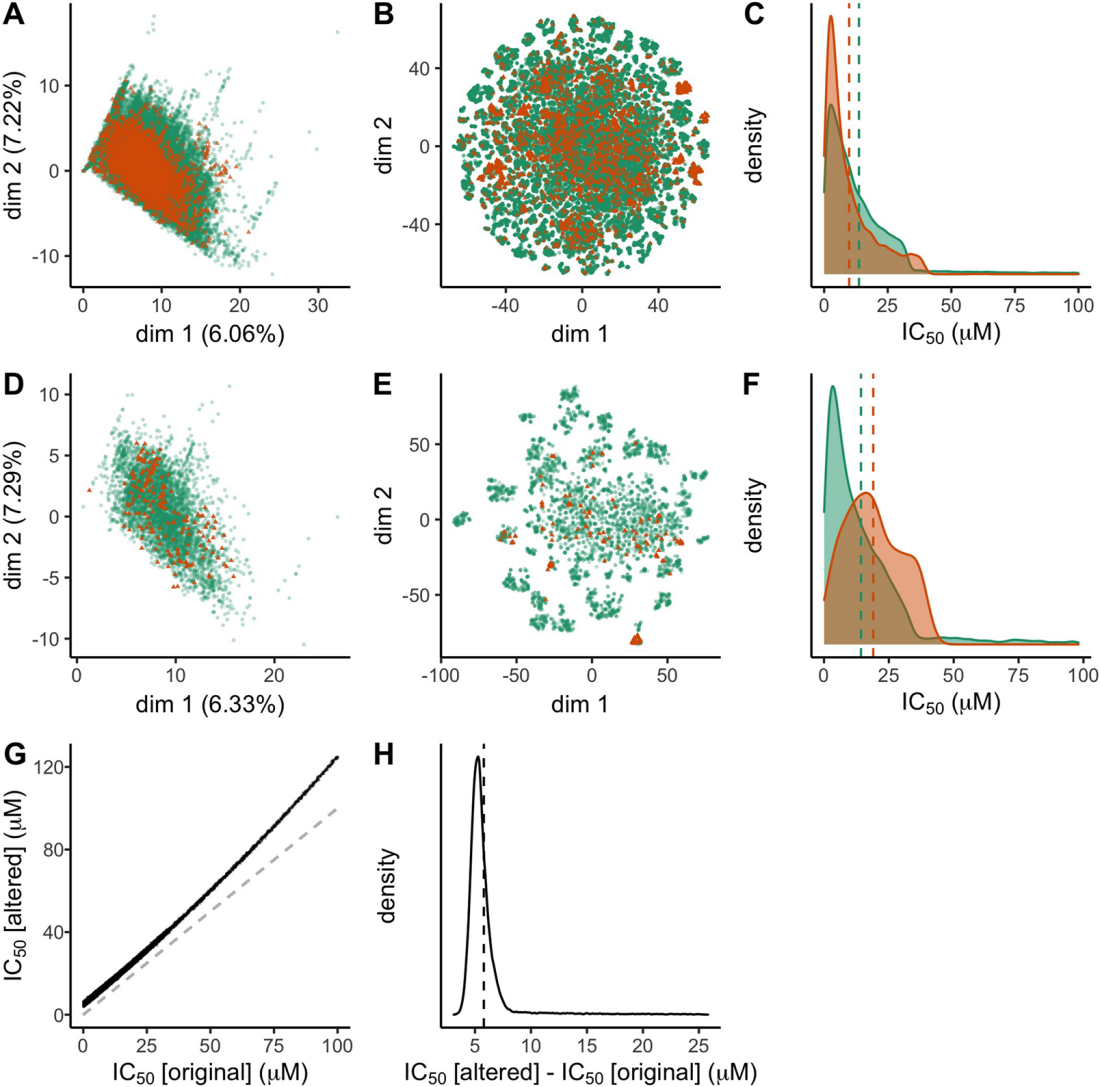
Visual analysis of the data sets. *A*_old_ is plotted in green, *A*_new_ is plotted in orange, the first row (A–C) shows hERG data, the second row (D–F) shows Na_V_, and the third row (G–I) shows the hERG augmented data set. Panels A and D plot the truncated SVD of the signature descriptors, explaining 13.28% and 13.62% of the total variance in data. Panels B and E plot the computed signature descriptors using t-SNE dimensionality reduction. Panels C and F plot the distribution of measured assay values, where the dashed lines show the mean for each assay. Panels G and H display the augmentation made to the hERG measurements in *A*_old_, expressed in IC_50_; the dashed line in panel G corresponds to a 1:1 relation between the original and augmented data (i.e., no change). The dashed line in panel H is the mean value of the plotted distribution.

Investigating potential divergences between the assays was also performed using the final data sets, in both descriptor space and the distribution of the measured assay values (Figure 2A–F). Truncated singular value decomposition (Truncated SVD) was used to compress the descriptor space into two dimensions, the score space was computed using the new assay data matrices (independently for hERG and Na_V_), and all records were then projected into the resulting score space (Figure 2A,D). Although only new data was used for computing the score space, there is an overall high similarity between the old and new assays. The old assays (green) are covering a larger area, suggesting that the diversity of compounds was higher for the old assays, or alternatively this could be a remnant of the sheer difference in the number of tested compounds, with the total explained variance of around 13% it is impossible to determine.

Figure 2B,E shows the result of t-SNE (t-distributed Stochastic Neighbor Embedding) dimensionality reduction computed using both old and new data combined (independently for hERG and Na_V_) after an initial step of truncated SVD into 50 dimensions. Similarly as for the truncated SVD, the old assay (green) covers a larger area compared to the new assay (orange). Here the records group into separate clusters, characteristic of the t-SNE algorithm, and not necessarily all due to true clusters in data. Nonetheless, panel E shows that many of the clusters are exclusively green (old assay records), and many of the orange triangles (new assay records) cluster together rather than spread uniformly with the green records. These clusters could be due to the typical drug discovery process, where in the lead optimization process many similar compounds are tested.^37^

Lastly, the distribution of the measured endpoint values from the assays was plotted in Figure 2C,F. Interestingly the difference between the old and new assays is the opposite for hERG and Na_V_, where the new hERG assay had a higher proportion of compounds with a stronger interaction and thus indication of a higher safety risk. For Na_V_, the change is the opposite, where the new assay has measured compounds with a lower degree of interaction. Possibly this difference could be due to the development process where the hERG assay is performed prior to Na_V_, and high risk compounds are excluded before further testing is performed.

The combination of the results shown in Figure 2A–F indicates that both hERG and Na_V_ assays are not i.i.d. between the old and new data, both in terms of descriptor space and the measured activity. Simply merging data from the legacy assay and the new assay would violate the requirement of i.i.d., and the resulting models would likely be unreliable.

### Augmented Data Set

Apart from the hERG and Na_V_ data sets, corresponding to real life data, an augmented data set was generated for simulating a scenario when transitioning between assays with less agreement. To keep the relevance to biological assays in the simulation, the augmented data set was constructed based on the hERG regression data sets. The descriptors were kept unchanged, while the assay measurements were altered with the goal of increasing the mismatch between the old and new assays. For hERG, the old assay reported compounds on average 2.54 μM (median 0.70 μM) higher than the new assay when analyzing the compounds measured in both assays (see Figure 1A). To increase the disagreement between the assays, the measurements of the old assay data were altered according to eq 1, where *G*_5,0.5_ denotes Gaussian random noise with μ = 5 and σ = 0.5.
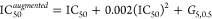
1

The transition of the augmented data set (altered old assay values) to the new hERG assay thus corresponds to a larger mismatch between the assay measurements compared to the original problem, see Figure 2G,H. The alteration includes a quadratic term in order to increase the mismatch for higher concentrations, as well as a randomized term that both performs a fixed shift and adds noise, resulting in an assay with different characteristics compared with the original.

### Conformal Prediction

Conformal prediction (CP)^21,25^ is a mathematical framework sitting on top of standard ML algorithms, proven to produce well-calibrated predictions adhering to user-defined *confidence* levels. To achieve this, the conformal predictor outputs *prediction intervals* (regression) or *prediction sets* (classification). A prediction is considered accurate if the true label is located within the interval or is part of the prediction set. Two types of conformal predictors, the transductive conformal predictor (TCP) and the inductive conformal predictor (ICP), are proven to produce well-calibrated predictions where the accuracy of the predictions is equal to or greater than the specified confidence the user asks for, given that data is *exchangeable*.^21^ Standard ML methods already impose the stricter requirement for data being i.i.d., so CP does not introduce any further requirements from what is already present.

Another group of inductive conformal predictors, collectively called Aggregated Conformal Predictors (ACPs), is based on training and combining several ICPs and merging their individual predictions into a single, final prediction. These are probably the most practically useful, with an improved informational efficiency (see the definition in the section Efficiency and Validity) compared to a single ICP,^38^ but do not retain the guaranteed validity which thus requires more effort to be put in validating the calibration of the resulting models.

#### Nonconformity

CP operates on the notion of *nonconformity*, or “strangeness”, of observations. The nonconformity of an object is calculated using a nonconformity function (or measure) which is typically derived from an underlying ML algorithm, and the nonconformity of test objects is what is used for generating the final prediction by a ranking against the nonconformity scores of a *calibration set*. For inductive conformal predictors, the type of predictors that is used herein, the calibration set is derived by sampling observations without replacement from the full training set. The remaining observations in the training set are called the *proper training set*, and these are used for training the underlying ML algorithm that is used in the nonconformity function. The nonconformity function is a parameter of the CP algorithm, and choosing a good function is key to optimal predictive performance.^39^

#### Efficiency and Validity

Conformal predictors are evaluated based on two concepts; *validity* and *efficiency*. Validity refers to the calibration of the predictions, verifying that the predictor adheres to the user-provided confidence level, and is typically confirmed with calibration curves where the accuracy is plotted against the desired confidence. Deviations from perfect calibration is, however, possible due to, e.g., test set size or calibration set size, and there is no strict method to definitively decide if a model is valid or not. The efficiency of a predictor quantifies the informativeness of the predictions and can be measured in many different ways,^40^ e.g., by the width of the prediction intervals (regression) or by the fraction of prediction sets that include a single label (classification). Compared with traditional model accuracy estimates for ML based on an external test set or cross-validation, where the same estimate is given to all test examples, CP delivers object-specific prediction intervals that depend both on the predicted object and on the user-defined confidence level.

### Study Design

The design of the experiments was aimed at benefiting readers in many scenarios of transitioning between assays, hence a large range of different sizes of *A*_old_ and *A*_new_ was evaluated, trying to simulate a wide range of possible combinations. After a transition to a new assay, the goal will be to predict the measurement that the new assay would generate for new compounds. Thus, testing was exclusively performed on data from the new assay. Each combination of *A*_old_ and *A*_new_ was evaluated with a 10-fold cross-validation (CV) of all the *A*_new_ data, repeated with ten replicates (*N* = 10). For each fold in the CV, the fixed number of samples was then drawn randomly from the training split of *A*_new_ from the CV and from the full *A*_old_ data set. Each replicate experiment had a fixed seed used for shuffling, CV splitting, sampling of data, and seeding the modeling algorithm, and the seeds were reused for all combinations of *A*_old_, *A*_new_, and a modeling strategy. Using this experimental setup facilitates the comparison of all results from the figures, as the test sets were identical at all points. All experiments using a specific *A*_old_ size (i.e., a single panel of the result plots) will have had access to exactly the same observations for training, and the same applies to a specific *A*_new_ size.

An artifact that comes with the study design is that the variance between replicates will become smaller as the data sizes increase, simply due to sampling more records out of the full data set will lead to more overlaps among the replicates. E.g., when using all observations in *A*_old_, the only difference between replicate experiments will be the splits of *A*_new_ in the CV, the sampling into calibration and proper training set in the CP algorithm, and the seed used for the modeling algorithm. We consider this to still be practically useful, even though statistically the plotted confidence intervals in the results are for the mean result of the population fixed to the complete data sets, rather than the mean for any possible set of compounds.

#### Evaluated Assay Transitioning Strategies

In this section, we describe the evaluated strategies for transitioning between two assays; a list with definitions can be found in Table 2. As mentioned previously, the ICP and TCP types of conformal predictors have been mathematically proven to be valid given exchangeable data. However, the TCP version is computationally demanding, as it requires retraining the underlying algorithm for every new prediction and is thus impossible for all but the smallest modeling problems. ICP, on the other hand, trains a single model using the proper training set, and this model is then used for all predictions, still producing valid models; but to some degree, reduced efficiency due to some training examples is set aside in a calibration set. One of the evaluated strategies was an ICP, termed ICP_old_^new^, where the proper training set was fixed to be all of the *A*_old_ data and the calibration set of all of the *A*_new_ data. Exchangeability is thus preserved between the testing data and calibration data, and it should thus be guaranteed to be valid and act at least as a reference point when inspecting the calibration curves, albeit presumably with lower efficiency than the other strategies.

**Table 2:** Modeling Strategies Evaluated in the Study^a^

strategy	aggregated models	proper training set	calibration set	exchangeable
CCP_new_	10	*A*_new_	*A*_new_	×
CCP_old_	10	*A*_old_	*A*_old_	
CCP_pool_	10	(*A*_old_ ∪*A*_new_)	(*A*_old_ ∪*A*_new_)	
ICP_old_^new^	1	∀*A*_old_	∀*A*_new_	×
CCP_AT_	10	*A*_new_ ∪ ∀*A*_old_	*A*_new_	×
CCP_AT2_	10	*A*_old_	*A*_old_ ∪ ∀*A*_new_	

a The *A* and *A* notation should be interpreted as 1 or 9 parts of a 10-fold split of the data set *A*, where the folds are shifted for each ICP model in a similar fashion as in cross-validation test-train splits. The last column indicates whether the model’s calibration set is exchangeable with the test data, i.e., theoretically guarantees valid models.

All remaining strategies were based on Cross-Conformal Predictors (CCPs),^41^ a type of ACP where data is randomly split into a calibration set and a proper training set in a folded fashion similar to *k*-fold cross-validation, consequently training *k* independent Inductive Conformal Predictors (ICPs), each with one fold for a calibration set and *k*–1 folds for a proper training set. The conformal p-values (classification) and intervals (regression) from the *k* predictions were aggregated using the median value, being the preferred method to retain good calibration of the final models.^42^ For the classification data set, the calibration was conducted in a Mondrian fashion, where the calculation of p-values is performed independently for each class, which has been shown to work well for imbalanced data sets without requiring under/oversampling, boosting, or similar techniques.^43,44^ Mondrian calibration was also performed in the ICP_old_^new^ modeling strategy.

The evaluated CCP-based strategies and their rationales were as follows (see also Table 2 for definitions):

- CCP_new_ which only uses *A*_new_ data and thus avoids potential issues of mixing data from different distributions.
- CCP_old_ which only uses *A*_old_ data, maximizing the number of training observations while avoiding mixing of data.
- CCP_pool_ which pools all *A*_old_ and *A*_new_ data, producing the largest data set, but potentially violating the i.i.d. assumption.
- CCP_AT_ which uses *A*_new_ data in the *k*-fold CCP splits but augments the proper training set of all ICPs by adding all *A*_old_ data–maximizing the amount of data in the proper training set but exclusively calibrating the predictions using observations from the new assay.
- CCP_AT2_ which uses *A*_old_ in the *k*-fold CCP splits and instead augments the calibration set of all ICPs by adding all available *A*_new_ data to the calibration set.

From these strategies, we expect the CCP_new_ and CCP_AT_ strategies to produce well-calibrated predictions, as the calibration sets are exchangeable with the test data which is only drawn from *A*_new_.

### Hyperparameters

A linear Support Vector Machine (SVM)^45^ was used as an underlying learning algorithm, successfully applied in previous QSAR studies in combination with the signature molecular descriptor,^46,47^ while being computationally less demanding compared to other kernel-based SVMs.^47^ The SVM *cost* parameter was set to 0.25, and ϵ (termed *p* in LIBLINEAR) in ϵ-SVR was set to 0.01. These parameters were found using grid-search of *cost* and ϵ without applying CP and instead optimizing the RMSE of the trained models. The full hERG regression data set, both exclusively using the new assay data and with a combination of both assays, and Na_V_, using only the new assay, were evaluated in the grid-search for *cost* and ϵ. The results in terms of RMSE were stable across the data sets, and the obtained *cost* value was close to an earlier benchmark study of SVM parameters,^48^ so no further tuning was conducted. The LIBLINEAR solver type was set to L2R_L2LOSS_SVR_DUAL for regression and L2R_L2LOSS_SVC for classification. The tolerance of the termination criterion was set to 0.001 following the default in the software that was used.

This study was conducted utilizing the implementation of ICP and CCP from the software CPSign version 1.5.0-beta4,^49^ with customized sampling strategies to match the sampling strategies of Table 2. The goal of the study was to compare different strategies of how the available data should be used, and improvements in absolute efficiency were of less interest. To facilitate the extensive number of experimental runs, no effort was put into finding optimal hyperparameters for each experimental setup; instead the *cost* and ϵ from the non-CP grid-search were used in all setups to give similar advantage/disadvantage to all setups. The number of folds in CCP (*k*) was set to 10.

The nonconformity function for classification was defined as the negative distance to the decision surface of the SVM (termed “NegativeDistanceToHyperplane” in CPSign). For regression, a normalized nonconformity function was used, which normalizes the prediction interval width depending on the predicted accuracy of the scoring model, following the definition in Papadopoulos and Haralambous^50^ (termed “LogNormalized” in CPSign). Both the scoring and error model were linear SVMs, with the nonconformity function outlined in eq 2, where α is the nonconformity score, *y* is the true label, *ŷ* is the predicted label from the scoring model, and μ̂ is the predicted error from the error model, all for instance *i*. The smoothing factor, β, was set to 0.01.
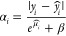
2

Linear interpolation of p-values and prediction intervals were used to accommodate for small calibration sets.^51,52^

### Code Availability

All essential code and instructions needed for running the experiments can be found in a GitHub repository at https://github.com/pharmbio/assay-transition-study. Note that the data preparation steps were left out as they were deemed too specific for the particular data sets, and the reader is instead referred to Box 1 and adapting it depending on their particular data.

## Results

The results are divided into subsections for each data set, and to simplify the interpretations, the models with insufficient calibration were excluded from the analysis in the paper; but all results can be found in the Supporting Information, alongside calibration plots for all experiments. As pointed out in the Conformal Prediction section, there is no definitive way to decide if a model is valid or not; here, we based our assessment on whether the calibration plot for the largest *A*_old_ size put the accuracy clearly below the expected accuracy to judge a modeling strategy to be invalid.

The result plots are arranged so that the reader can pick the *A*_old_ size that is of interest (i.e., picking one of four panels) and follow the trends of what happens when more data is generated with the new assay (i.e., changes along the *x*-axis). Efficiency is plotted using the 95% confidence intervals (CI) of the mean result values assuming a t-distribution, computed from the 10 replicates for each experiment combination (*A*_new_ size, *A*_old_ size, and strategy). As described in the Study Design section, the CI will become smaller for larger *A*_new_ and *A*_old_ sizes, due to training data having larger overlaps of observations as more data is sampled.

### hERG Classification Data Set

**Figure 3:**
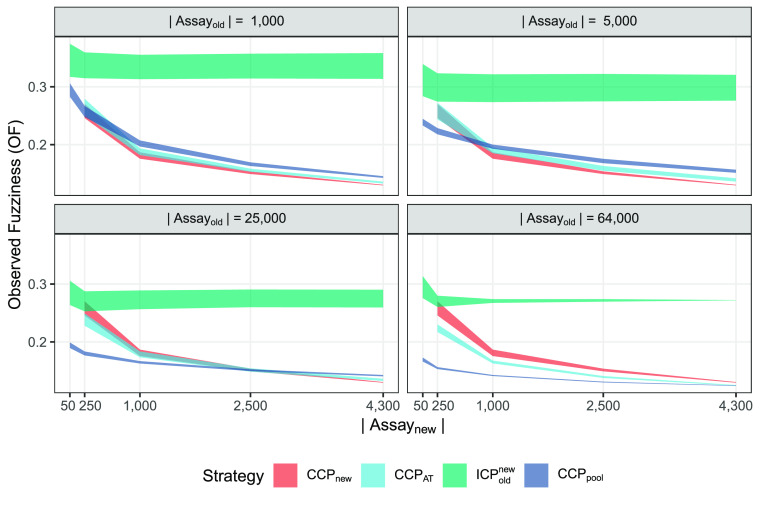
hERG classification results for all well-calibrated strategies, plotting the Observed Fuzziness (smaller values are better). The colored areas correspond to the 95% confidence intervals computed from the ten replicate runs. Note that the CCP_new_ strategy is independent of *A*_old_ size and will thus be the same in all four panels. The overall winning strategies are CCP_pool_ and CCP_AT_, depending on the combination of *A*_new_ and *A*_old_ size, shifting from CCP_pool_ to CCP_AT_ when the number of compounds in *A*_new_ exceeds 10% of that of *A*_old_.

hERG classification was the largest data set, and it was evaluated using Observed Fuzziness (OF) as efficiency metric. OF is a confidence-independent metric, which is favorable as the desired (or required) confidence can vary between use cases. OF is calculated using the average sum of the p-values for the nontrue classes; following Vovk et al.,^40^ smaller values are preferable. The results are shown in Figure 3, where the 95% CIs are plotted with colored ribbons. Only strategies CCP_new_, CCP_AT_, and ICP_old_^new^ produced well-calibrated models for all setups, whereas CCP_pool_ produced valid models for the three smallest *A*_old_ data sets and were slightly below the desired accuracy when using all *A*_old_ data (see Supporting Information Figure S1 for all calibration plots). It was thus included, even though borderline invalid.

Strategies CCP_new_ and CCP_AT_ were removed when using *A*_new_ only contained 50 observations, as the calibration sets became too small (10-fold CCP uses 10% of all data as the calibration set, i.e., only five observations out of two different classes, which was not allowed in the CP implementation that was used).

The overall trend is that the efficiency improves as the number of training observations increases, which is expected. The ICP_old_^new^ strategy is the worst in terms of efficiency, consistent with literature and the reason for using ACPs. CCP_new_ and CCP_AT_ are very similar in terms of OF and have overlapping CIs in many cases, with only a clear separation in favor for CCP_AT_ when including all *A*_old_ data. Analyzing the four panels jointly, the overall most efficient strategy is to use the CCP_pool_ when having more than ten times as many compounds in the *A*_old_ assay and then start to use CCP_AT_ (the CCP_pool_, CCP_new_, and CCP_AT_ overlap at 500, 2,500, and 4,300 for panels 2–4). If requiring strictly well-calibrated models, the CCP_pool_ has to be excluded in panel 4, making CCP_AT_ the preferred strategy in that case.

### hERG Regression Data Set

**Figure 4:**
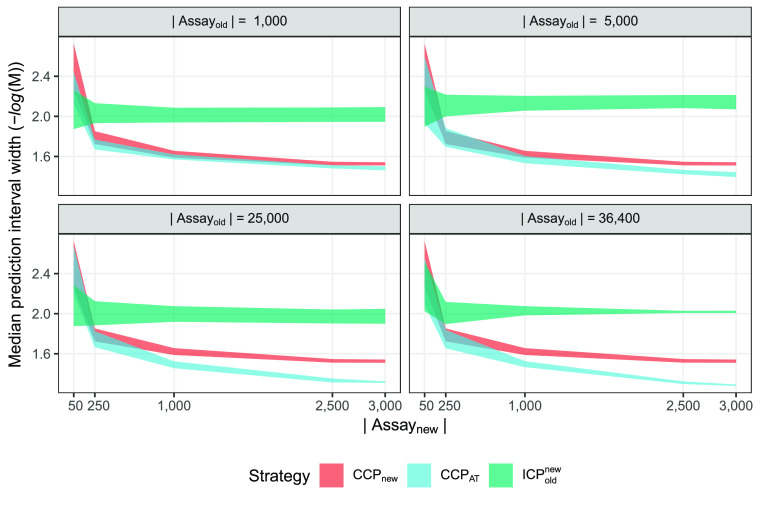
hERG regression results for all valid models. Efficiency is expressed in terms of prediction interval width at a fixed confidence of 0.8; smaller values are preferable. The colored areas correspond to the 95% CI computed from the ten replicate runs. The overall winning strategy is the CCP_AT_, having overlapping CIs for the smaller *A*_new_ sizes but favorable when more data is used from *A*_new_. The results are more prominent in the second row of panels, where more *A*_old_ observations are used.

The hERG regression data set was slightly smaller than the corresponding categorical data set. Efficiency was computed as the median prediction interval width at a fixed confidence of 0.8, expressed in pIC_50_ (negative log molar concentration). The choice of 80% confidence was based on domain and data set knowledge, as the data was known to have too much variance and noise for realistically expecting informative results at a higher confidence. For this data set and the following regression data sets, only three strategies produced well-calibrated models in all experimental setups: CCP_new_, CCP_AT_, and ICP_old_^new^. The results are shown in Figure 4, plotting the 95% CI for the ten replicate runs.

Similarly as for the classification data set, the ICP_old_^new^ strategy was overall inferior in terms of efficiency. The only scenario where ICP_old_^new^ is preferable was for the smallest *A*_new_ size, explainable by the size of the calibration set where CCP_new_ and CCP_AT_ only have five observations and the ICP_old_^new^ can use all 50 observations for calibration. The overall best strategy was CCP_AT_, especially in panels 3–4 with more available data from *A*_old_.

Compared to the classification setting, the CCP_pool_ strategy was mostly invalid (see the Supporting Information), having a lower accuracy than the desired confidence. In the calibration plot in Figure S2, it looks like the CCP_pool_ is overconservative for the smallest *A*_old_ data set and thus a valid strategy, but when reviewing the calibration for each combination of *A*_old_ and *A*_new_ size (data not shown), it was evident that valid models were only produced when *A*_new_ made up at least 20% of the total training set (i.e., the two largest *A*_new_ sizes, where CCP_pool_ was less efficient than both CCP_AT_ and CCP_new_). Thus, the CCP_pool_ either produced invalid models or was outperformed by other strategies.

### Na_V_ Regression Data Set

**Figure 5:**
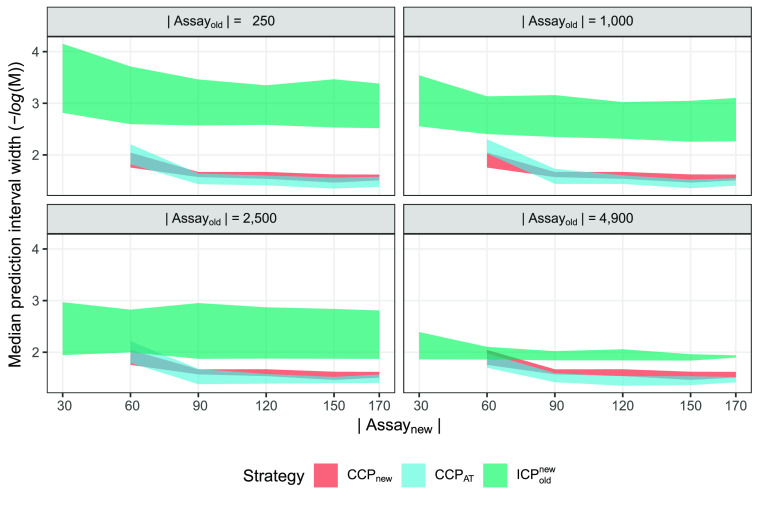
Na_V_ regression results for all valid models. Efficiency is expressed in terms of prediction interval width at a fixed confidence of 0.8; smaller values are preferable. The colored areas correspond to the 95% CI computed from the ten replicate runs. The ICP_old_^new^ was the only possible strategy to use when only having 30 available compounds in *A*_new_ but was surpassed in efficiency in all other experimental setups. CCP_AT_ and CCP_new_ have very similar efficiencies, and there is no clear winning strategy.

The Na_V_ data set was the smallest data set, only containing 190 tested compounds in the new assay, possibly making it the most interesting and potentially beneficial to include additional data in the modeling. Results are shown in Figure 5, again using median prediction interval width at confidence 0.8 as the efficiency metric, expressed in units of pIC_50_. Similar to hERG, the three strategies CCP_new_, CCP_AT_, and ICP_old_^new^ were the only ones producing well-calibrated models at all experimental setups. The CCP_pool_ was again mostly invalid, but for panel 1, with the least amount of *A*_old_ data, it was producing valid predictions and thus would be the preferred strategy when only having 30 compounds in *A*_new_ and then getting surpassed by the CCP_new_ and CCP_AT_ (Figures S3 and S7).

The results are similar to those found for the hERG Regression Data Set section but less pronounced. CCP_AT_ and CCP_new_ are in most cases overlapping in terms of efficiency, with possibly CCP_AT_ improving the efficiency compared to CCP_new_ with more data in the old assay, but nothing definitive can be said. Interestingly, the ICP_old_^new^ improves in efficiency with the inclusion of more *A*_old_ data, more so than for the hERG experiments; extrapolation of the results from all panels indicate that there could be a scenario where this strategy could become better than the other two, if more *A*_old_ data were available.

### hERG Augmented Data Set

**Figure 6:**
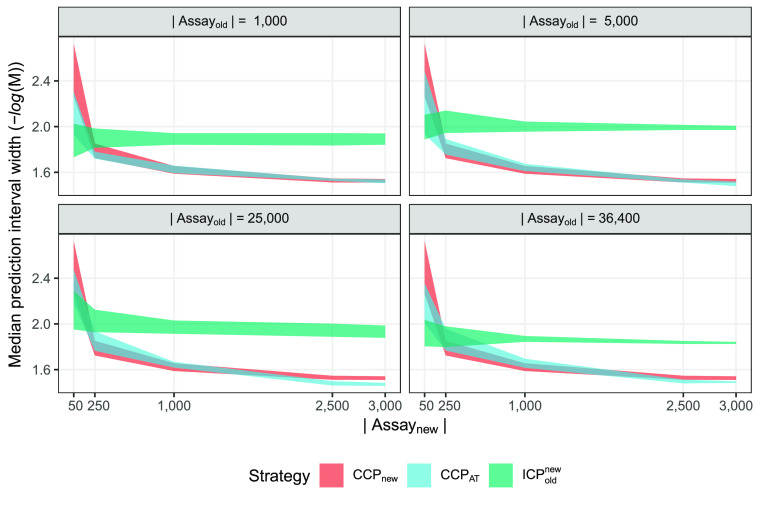
hERG augmented data set results for all valid models. Efficiency is expressed in terms of prediction interval width at a fixed confidence of 0.8; smaller values are preferable. The colored areas correspond to the 95% CI computed from the ten replicate runs. The overall results are similar to those in Figure 4 but with CCP_AT_ performing slightly worse here. CCP_AT_ and CCP_new_ strategies are mostly overlapping but with CCP_AT_ having a small advantage in the last two panels and the two largest sizes of *A*_new_.

For the augmented data set, simulating a larger divergence between assay measurements by altering the labels of the *A*_old_ observations, the evaluation was performed in the same way as for the hERG regression data set experiments, and the results are shown in Figure 6. Comparing these results to those of the unaltered data (Figure 4) makes it possible to assess the usefulness of additional data even when there is less agreement between the assays. Note that strategy CCP_new_ is identical to those of the unaltered experiments as it uses no augmented data.

The results are similar to the unaltered experiments, but the improvement in efficiency of CCP_AT_ over CCP_new_ seen in the previous experiment is less pronounced and only found when including 2,500 or all of the *A*_new_ data. Another interesting difference is that the ICP_old_^new^ had an improved efficiency compared to the unaltered experiments and is arguably the best strategy when only having 50 records in *A*_new_.

## Discussion

Over time as new assays are incorporated and old ones are phased out, organizations are faced with challenges on how to maintain predictive models that are valid and as accurate as possible. If the accumulated data from old assays is small in relation to the data generation in the new assay, this might not constitute a big problem. However, if the organization has invested significant efforts in building up a knowledge base for predictive modeling based on one type of assay, it would be highly profitable to maximize the usefulness of this data after transitioning to a new assay–especially if it will take some time for the accumulated data from the new assay to reach levels where models with high accuracy can be trained. It is generally advised to perform experiments to assert assay concordance between the old and new assays before deciding and implementing a change; but once a new assay has been implemented, the problem still remains as to how new machine learning models should be trained on data from both the old and new assays. Herein, we investigated modeling strategies based on conformal prediction in order to produce valid (well-calibrated) models with the highest informational efficiency.

The overall trends in the results are consistent across the four data sets, with the exception of the CCP_pool_ modeling strategy which was producing valid models for most runs for the classification experiments (Figure 3), whereas for the regression data sets, it was invalid. Out of the remaining strategies, the three modeling strategies CCP_new_, CCP_AT_, and ICP_old_^new^ were found to always be valid, which can be linked back to the discussion in the section Conformal Prediction, as these three strategies are the only ones that theoretically would be valid according to standard conformal prediction proofs (disregarding the potential invalidity of CCP and variances in calibration due to the finite number of test samples). These conflicting results are likely due to classification problems being typically easier to model than regression problems but nevertheless make the CCP_pool_ a strategy necessary to evaluate as it had cases with a clear advantage over the other strategies in terms of efficiency.

Between the regression experiments, the overall best strategy was to use ICP_old_^new^ when there is an insufficient amount of data in the new assay (50 examples for hERG and 30 for Na_V_) to produce efficient models and then start to use either CCP_new_ or CCP_AT_. Overall, there are no scenarios where the CCP_new_ is preferred over the CCP_AT_, as their CIs either overlap or CCP_AT_ is superior. Comparing the augmented data set experiments with the unaltered experiments demonstrates that there is less advantage of the CCP_AT_ when there is a larger discrepancy between the assays, where CCP_AT_ is only favorable in the scenarios with access to a great deal of data from both the old and new assays. For the classification data set, these three strategies had similar trends as for the regression data sets, but the CCP_pool_ strategy was clearly preferable in scenarios with access to at least a 10-fold excess of *A*_old_ observations over that of *A*_new_.

Our overall objective was to demonstrate and evaluate several different scenarios of transitioning between assays and how to use data from a legacy assay with conformal prediction. The results show that the best possible usage of old data depends both on the amount of data each assay has and agreement between the assays. Figures 3–6 for hERG and Na_V_ assays allow for discussing different practical scenarios relating to size of old and new assays and assay concordance. A key finding is that the best possible strategy can change while gathering more data using the new assay, and we therefore propose that the way data from the old assay is used should be evaluated continuously when data from the new assay is produced, instead of performing a single evaluation and then persisting with that strategy indefinitely. Such one-time evaluation could potentially result in a suboptimal strategy in the long term. The results in our study show that a simple strategy such as pooling old and new data might lead to invalid models, even if the assays are deemed to have enough concordance. Further, we show that training models based only on new data will lead to less efficient models until enough data has been produced.

Classification is normally a simpler task than regression, and when comparing Figures 3 and 4, we see that the value of old data and our proposed assay transition modeling strategy (CCP_AT_) is larger in the case of regression compared to classification. Further, comparing the results in Figure 3 with Figure 6 shows that the value of data from the old assay is lower when it is disturbed (hence a lower assay concordance), and the benefit of the CCP_AT_ strategy is also lower.

A benefit of using conformal prediction compared to traditional ML is that the calibration of the predictions can be monitored alongside the efficiency metrics, making it possible to discover data drifts or improper handling of data (e.g., by pooling data incorrectly). Although we point out that there is no absolute way to determine the validity of a model in a strict sense, deviation from perfect calibration can occur due to the finite number of test examples (making statistical fluctuations have an impact) and choice of predictor type.

One assumption that was made in this study was that the goal is to predict the outcome of the new assay, exclusively evaluating performance on observations from the new assay. We argue that this is preferable as new experiments will only be conducted in the new setup. We also expect a natural drift in the tested compounds (i.e., exploring new regions of chemical space), making compounds tested more recently being more relevant for future projects. The evaluation will thus be more useful compared to performing the evaluation of pooled data from both assays, but we expect the validity of the strategies to be very much affected based on the testing strategy.

To facilitate the large number of evaluated scenarios, minimal parameter tuning was performed, which could have some effects on the results as the SVM *cost* and ϵ parameters were determined using all available data which could be suboptimal for the smaller data set sizes. Another angle of investigation would be to replace the linear SVM with a more complex learning algorithm, such as an RBF kernel SVM, in those cases where it is feasible with respect to run time. This alternative strategy could lower, e.g, the benefit of using CCP_AT_ over CCP_new_ in scenarios where a great deal of old data is used, making the CCP_AT_ infeasible to train using an RBF kernel, while practically possible when only using new data. This, however, is arguably a more data set dependent analysis and thus not pursued herein but could potentially have an impact in some situations.

## Conclusions

We show that it is important to continuously monitor predictive models when transitioning between assays, in order to maximize the usefulness of data from the old assay. We suggest to use conformal prediction in this transitioning process to measure both the level of calibration in addition to efficiency of models, thereby ensuring that invalid models can be identified and dismissed. We also propose a modeling strategy where data from the old assay is used to expand the proper training set of an inductive conformal predictor and where calibration is performed exclusively on data from the new assay, resulting in valid models and the best overall efficiency across all experiments.

## Supporting Information Available

Calibration and efficiency plots for all modeling strategies and all data sets (PDF)

## Notes

**Data and Software Availability**. The data analyzed in this study was provided by AstraZeneca and is proprietary. No publicly available data sets from single experimental setups exist of this magnitude, thus forcing the study to be performed with proprietary data. We emphasize that our key findings are not dependent on the particular data or the obtained predictive efficiency, instead it shows the importance of monitoring changes and the potential usage for conformal prediction to resolve issues related to data set shifts. In an effort to validate our findings on public data, we present an example using data from the Tox21 Data Challenge,^53^ found at https://github.com/pharmbio/assay-transition-study. However, the Tox21 data was acquired using the same experimental setup in their separate data sets, translating to a different type of data set shift compared to what is studied in this paper, thus only serving as a crude validation. The CPSign software used in this study is proprietary; but a free Academic license can be acquired, and all code needed to run similar experiments is freely available in a GitHub repository at https://github.com/pharmbio/assay-transition-study.
