# Supplementary material for: Machine Learning Strategies When Transitioning between Biological Assays
Source: J Chem Inf Model. 2021 Jun 21;61(7):3722–33. doi: 10.1021/acs.jcim.1c00293 (PMC8317157; doi:10.1021/acs.jcim.1c00293)
Supplement: Supplementary file 1 — ci1c00293_si_001.pdf [file ci1c00293_si_001.pdf]

Supporting Information
Machine learning strategies when transitioning between biological assays

Staffan Arvidsson McShane,[*,†] Ernst Ahlberg,[†,‡,§] Tobias Noeske,[¶] and Ola Spjuth[†]

†*Department of Pharmaceutical Biosciences and Science for Life Laboratory, Uppsala University, 751 24 Uppsala, Sweden*

‡*Stena Line Scandinavia AB, AI & Data, 405 19 Gothenburg, Sweden*

¶*Imaging and Data Analytics, Clinical Pharmacology & Safety Sciences, R&D, AstraZeneca, 431 50 Gothenburg, Sweden*

§*Previous address: Predictive Compound ADME & Safety, Drug Safety & Metabolism, AstraZeneca IMED Biotech Unit, 431 50 Gothenburg, Sweden*

E-mail: staffan.arvidsson@farmbio.uu.se

The supplementary data contains full calibration plots, for all A_{old} sizes and all six evaluated modeling strategies (Figure S1-S4), as well as result plots for all six strategies (Figure S5-S8). The calibration plots are divided column-wise and ordered left to right according to the number of compounds used from A_{old}, where the right most plots are for using all available old data. Also note that the plots for CCP_{new} modeling strategy are identical in all columns, as no old data is being used. Similarly as in the manuscript, the plots are plotting the 95% confidence interval for all runs using the same combination of A_{old} and A_{new} data.

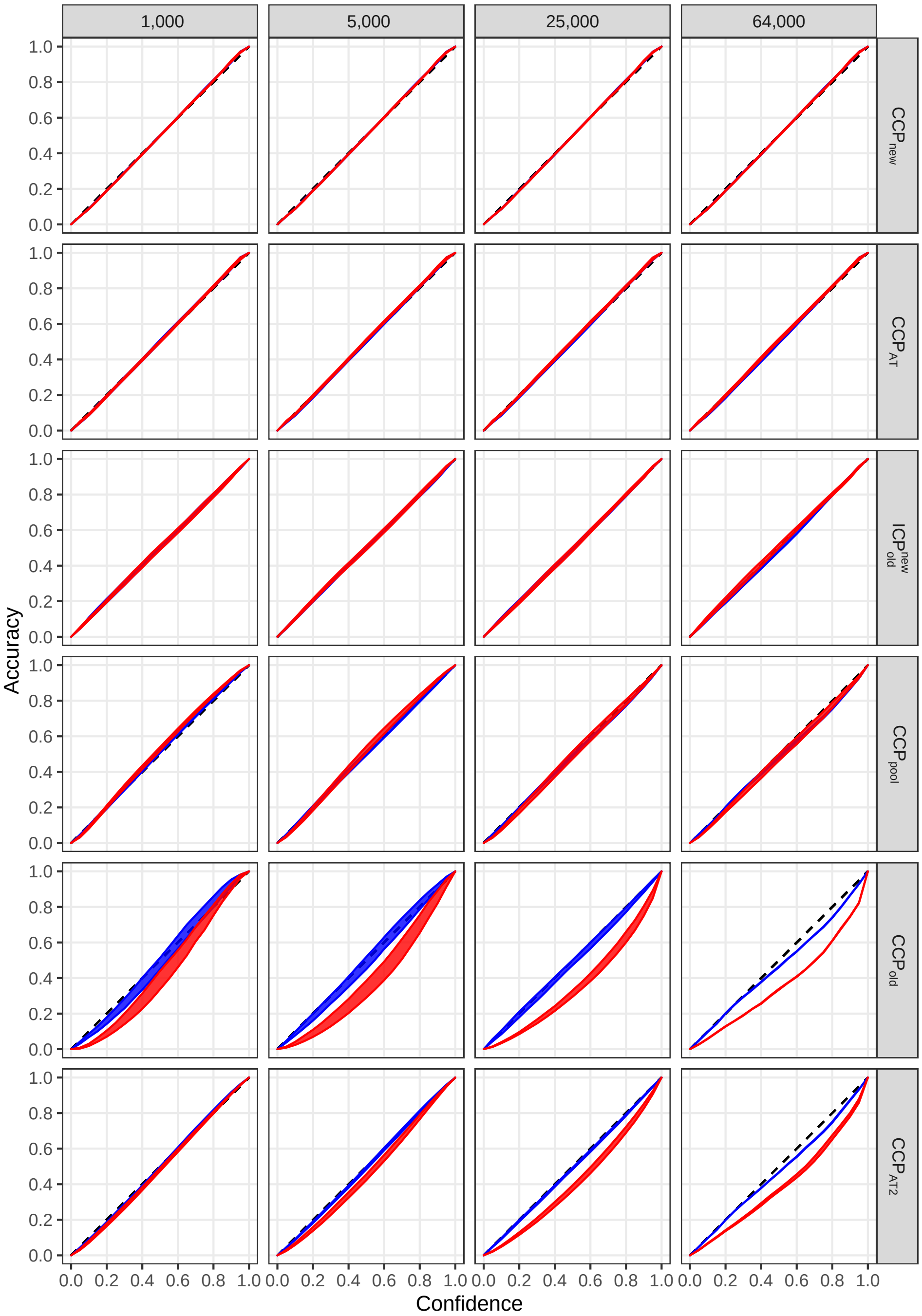

Figure S1: Calibration plots for hERG classification for all six strategies. Here the calibration is plotted for each class independently, active plotted in blue and non-active plotted in red. Strategies CCP_{new}, CCP_{AT} and $\text{ICP}^{\text{new}}_{\text{old}}$ all have calibration curves that indicate valid models, i.e. observed accuracy roughly corresponds to the desired confidence. CCP_{pool} is conservatory valid, i.e. slightly higher accuracy then set confidence, in the left most plot and produce slightly lower accuracy then desired confidence when using all available data (the right most plot). Strategies CCP_{old} and CCP_{AT2} has the worst calibration, even though CCP_{AT2} is close to valid in the two left-most plots.

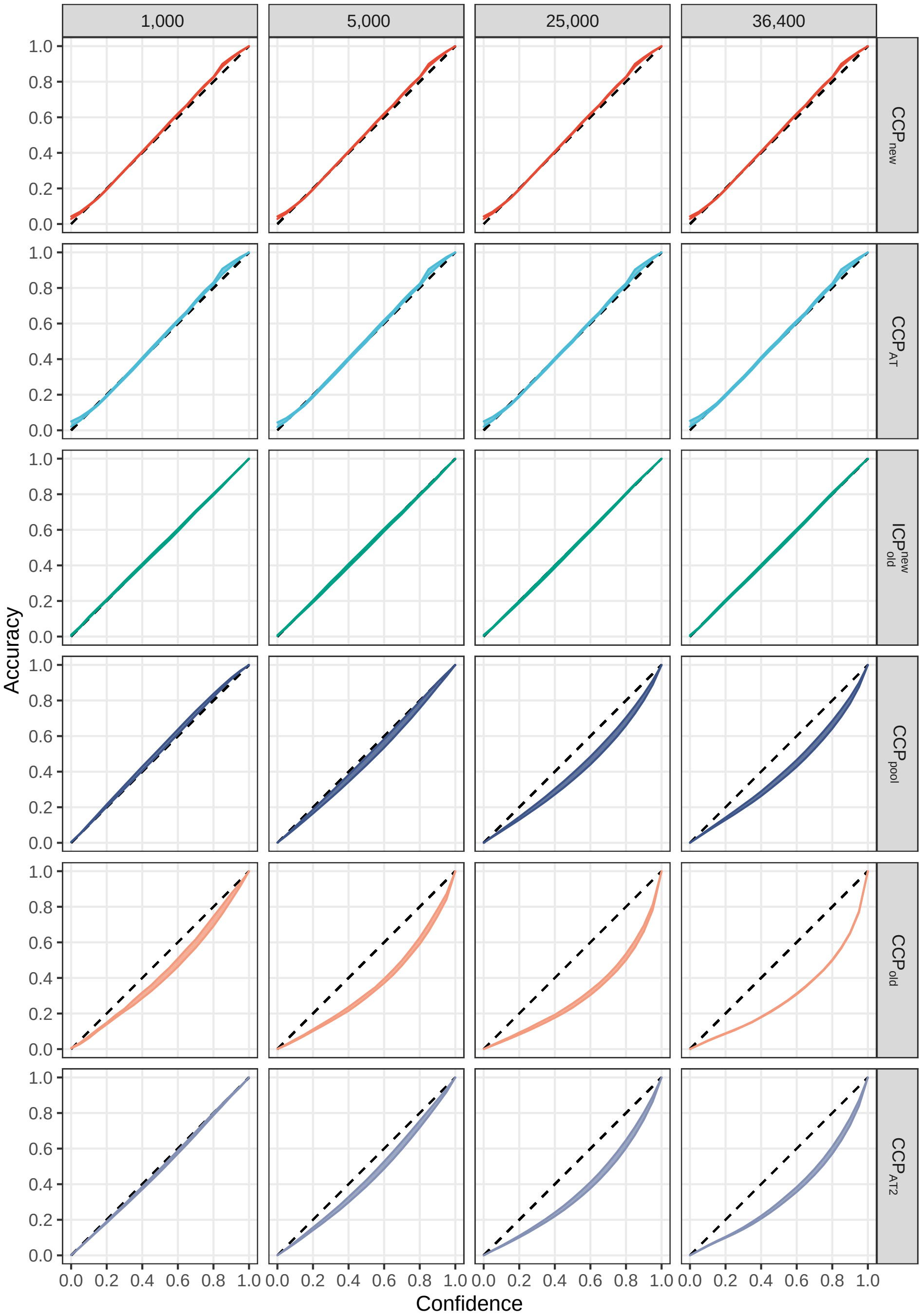

Figure S2: Calibration plots for hERG regression for all six strategies. Comparing with the plots in Figure S1 these plots show a similar pattern, where $\mathrm{CCP}_{\mathrm{new}}$, $\mathrm{ICP}^{\mathrm{new}}_{\mathrm{old}}$ and $\mathrm{CCP}_{\mathrm{AT}}$ all have calibration curves that indicate valid models. The calibration curves for the remaining strategies are worse than for the classification case, where $\mathrm{CCP}_{\mathrm{old}}$ and $\mathrm{CCP}_{\mathrm{pool}}$ produces invalid models already when including 5,000 of the old compounds and $\mathrm{CCP}_{\mathrm{AT2}}$ deviates even more from validity.

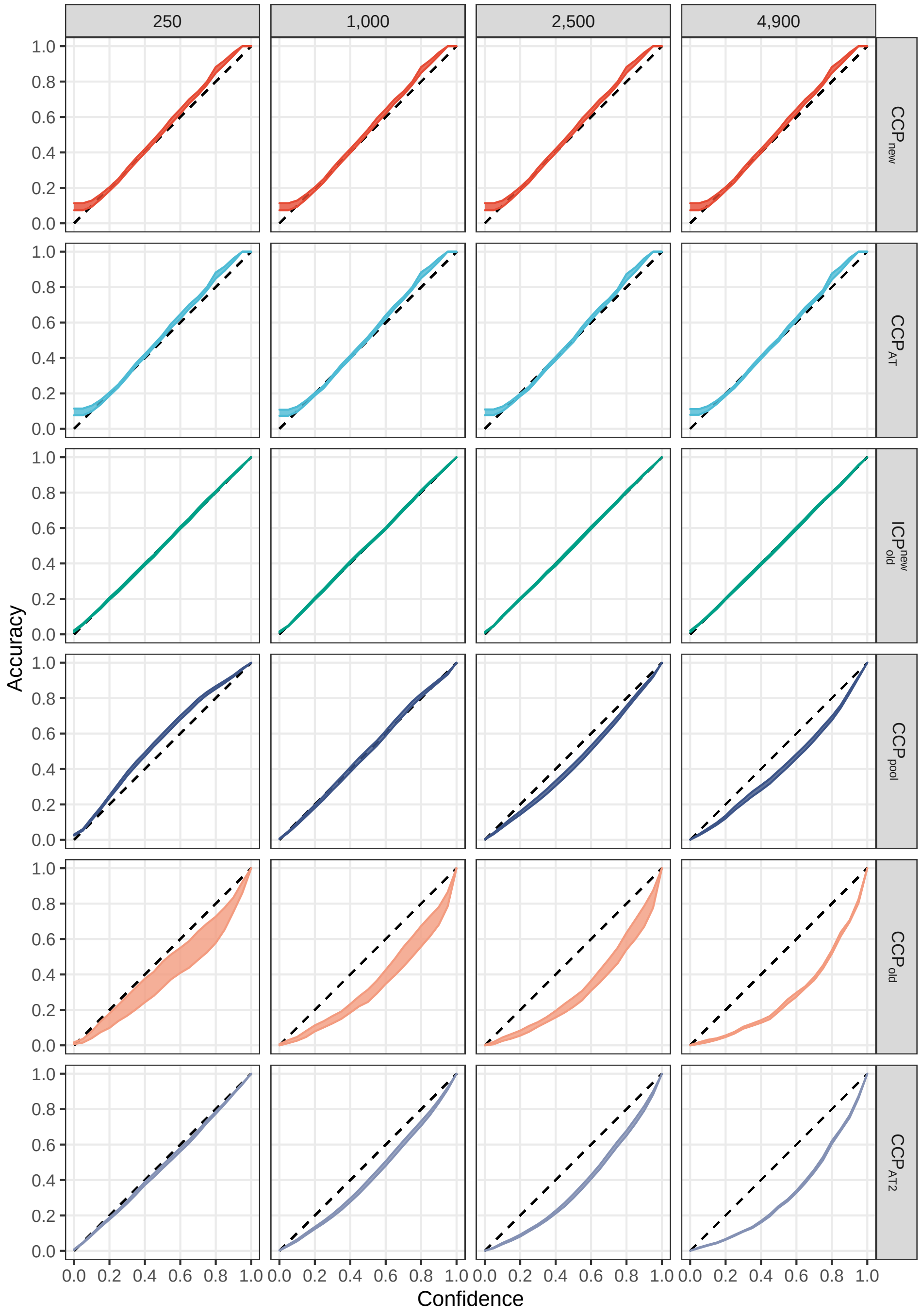

Figure S3: Calibration plots for Na_V regression for all six strategies. Similarly as for hERG, the same three strategies, CCP_{new}, ICP^{new}_{old} and CCP_{AT}, all have calibration curves that indicate valid or conservative valid models. CCP_{pool} is conservatory valid for the smallest A_{old} data size, valid for the 1,000 A_{old} and then produce invalid models. CCP_{AT2} produce valid models for the smallest A_{old} data size and then produce invalid models that deviates greatly from set confidence when including 2,500 and all compounds from A_{old}. CCP_{old} produce invalid models for all sizes of A_{old}.

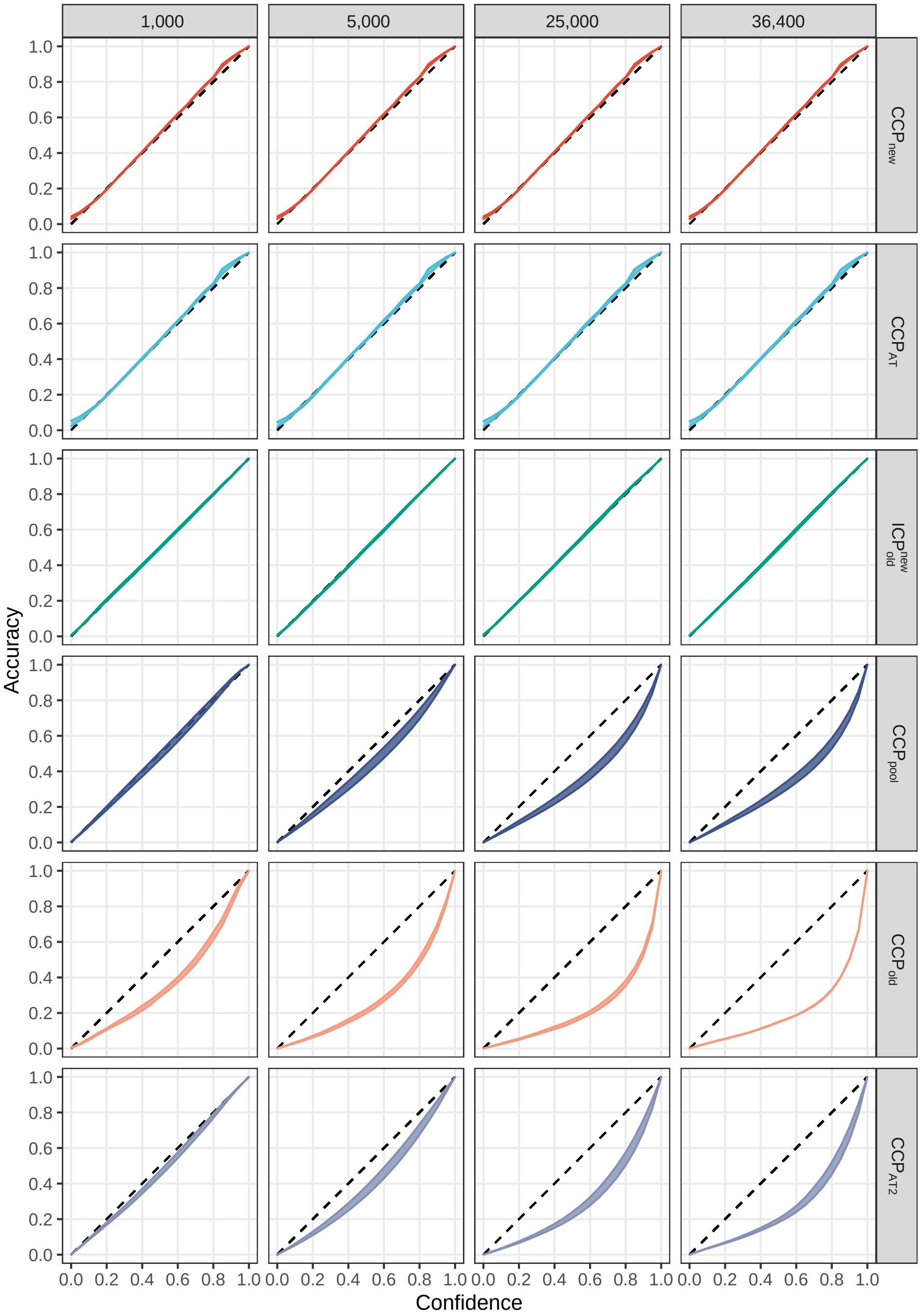

Figure S4: Calibration plots for the augmented data set for all six strategies. Similarly as for the three other data sets the same three strategies, CCP_{new}, $\text{ICP}^{\text{new}}_{\text{old}}$ and CCP_{AT}, all have calibration curves that indicate valid models. CCP_{pool} strategy is valid when including only 1,000 of $\text{A}^{\text{aug}}_{\text{old}}$ compounds and then produce invalid models. CCP_{old} and CCP_{AT2} produce invalid models for all sizes of $\text{A}^{\text{aug}}_{\text{old}}$.

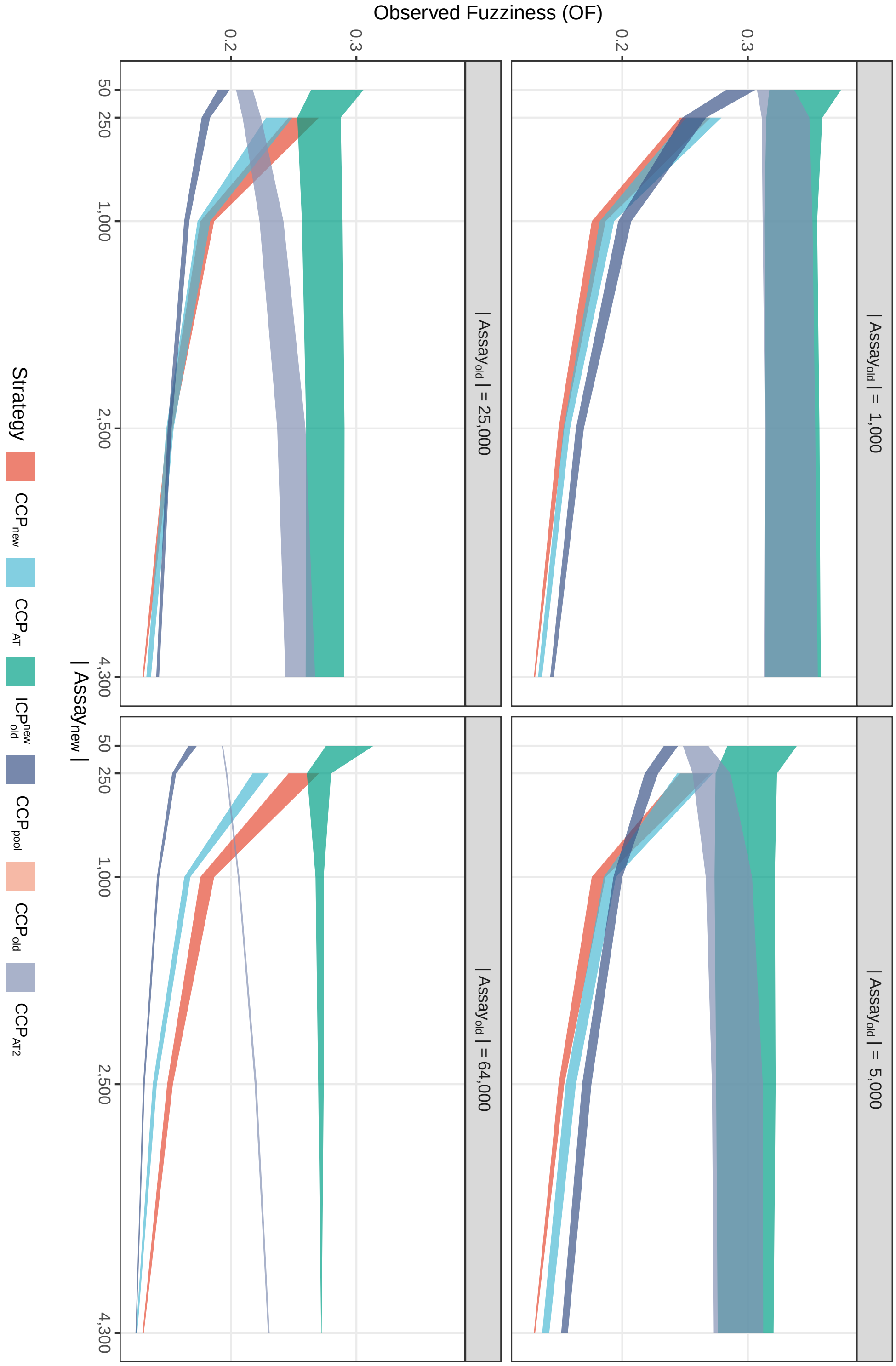

Figure S5: hERG classification results for all six strategies.

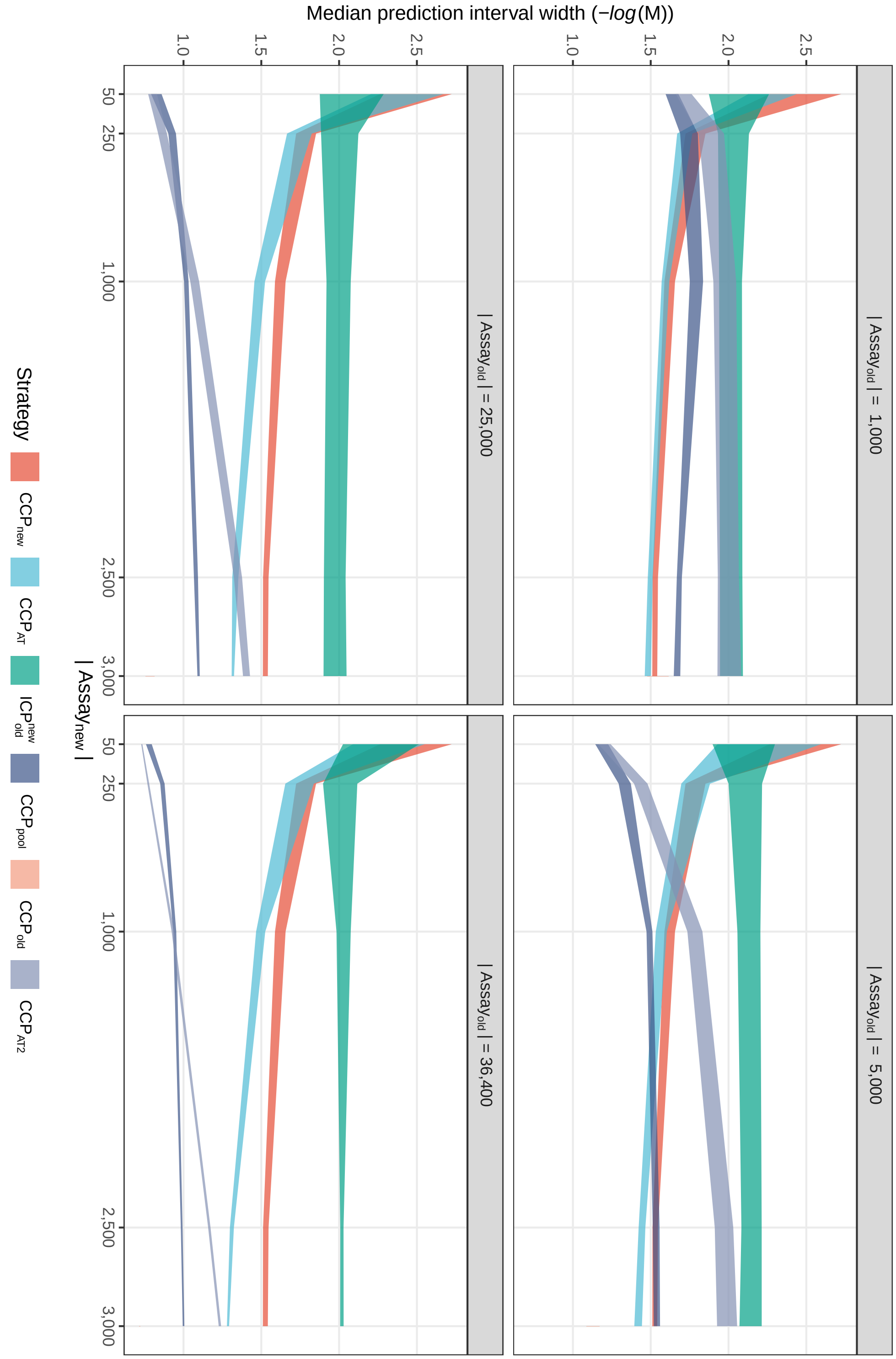

Figure S6: hERG regression results for all six strategies.

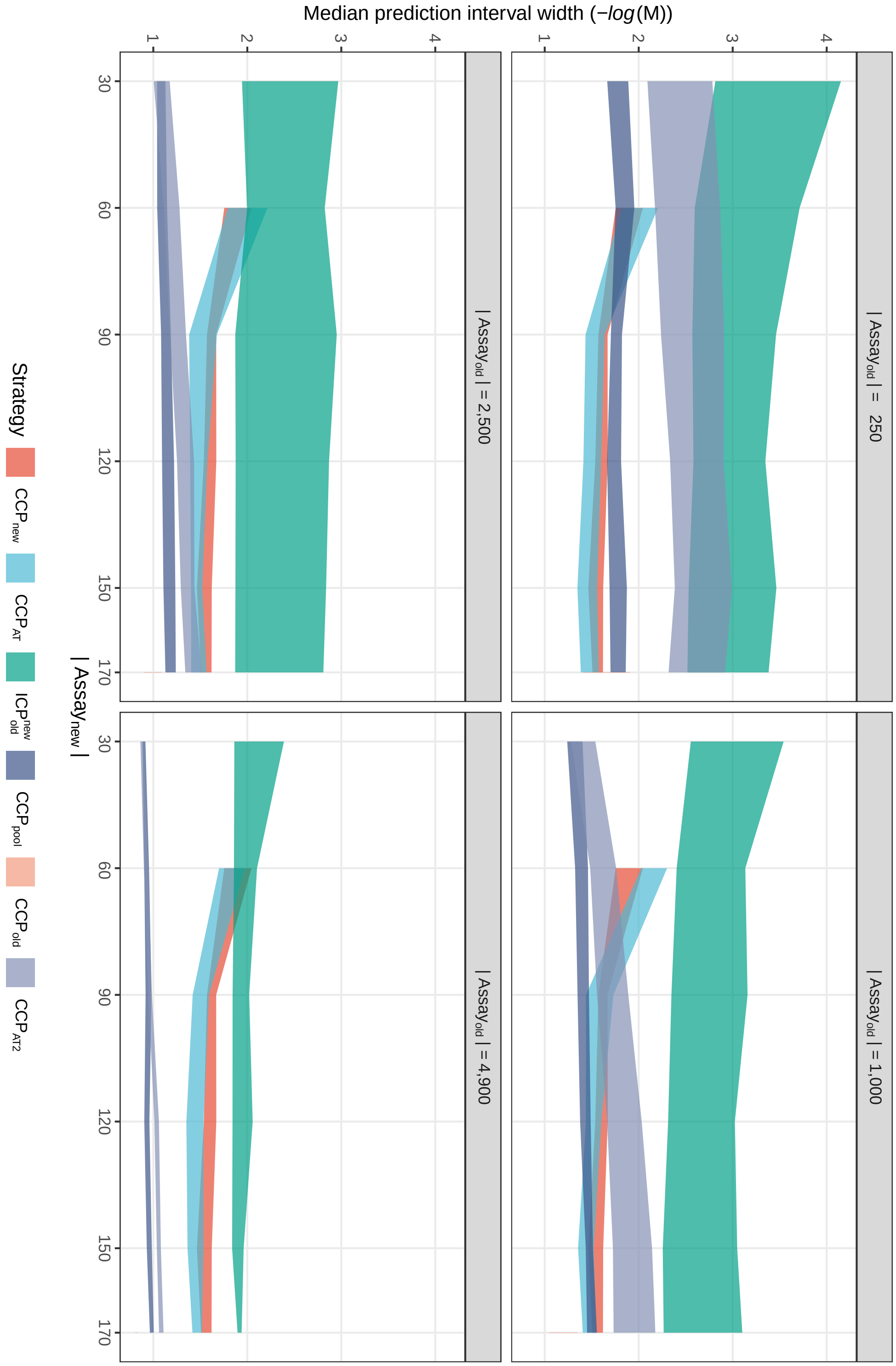

Figure S7: Na_V regression results for all six strategies.

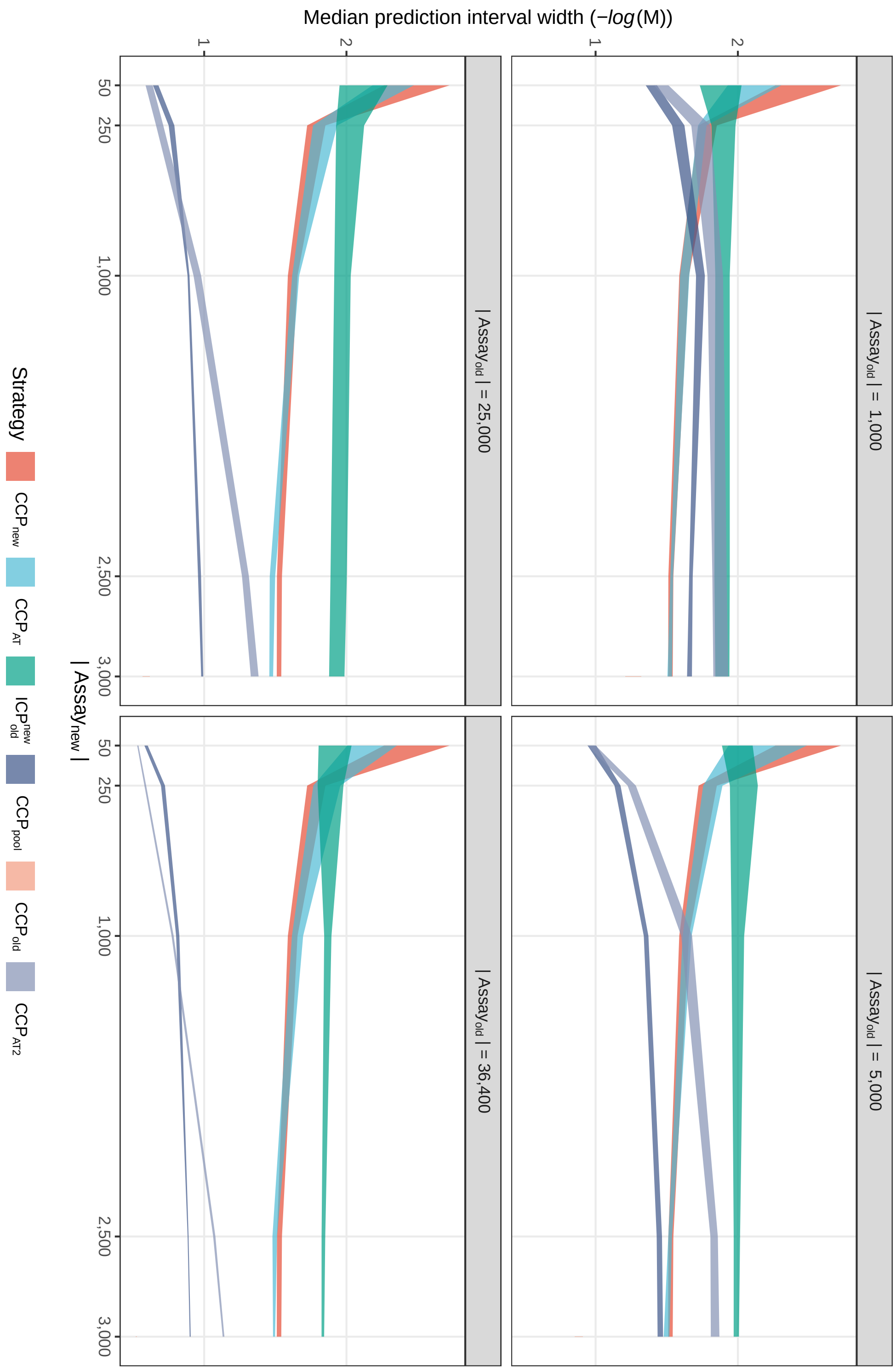

Figure S8: Augmented data set results for all six strategies.
